# Surgical Cases

**Published:** 1853-11

**Authors:** George W. Brown

**Affiliations:** Port Carbon, Pa.


*Surgical Cases.* By GEORGE W. BROWN, M. D., of Port Carbon, Pa.

(Continued from No. VII.)

CASE IV. Jonathan D., a stable-man, fifty years of age, five feet ten inches high, pretty well made, but of rather dissolute habits, came into my office November 9th, 1852, with his arm supported in a sling, and desired me to examine his shoulder. I stripped him, and at a single glance, I saw he had a dislocation of the right os humeri into the axilla. The shoulder was flattened, the acromion process of the scapula very prominent with a depression immediately under it, the arm was immoveably fixed, and the head of the bone could be felt in the axilla.

The history he gave of his case was that two weeks previous he went to Pottsville for a load of feed, and he drank rather freely, and when he came home he opened one of his bags, and tóok hold of the mouth with his right hand, and placed it upon his right shoulder, and in attempting to walk a plank from his waggon to the stable with his load, he was precipitated to the ground a distance of three feet, and as he fell he felt something give way in his shoulder, and when he arose he suffered severe pain in the part, and found the arm immoveably fixed. He immediately called in a neighboring physician, who came, and made extension for some time, and finally told him it was reduced. He placed a pad in the axilla, put the arm in a sling, and then forced the elbow as close to the side as he could with a bandage placed around the arm and body, and left. He called the next day, and as he was suffering acute pain he insisted the bone was not in its place, but the Doctor gave him a liniment to rub his shoulder, assuring him that it was reduced; and thus things stood when he came to me, the pain never ceasing in his shoulder for one moment. I placed him in a chair and applied a proper apparatus, and after torturing my patient for some time, and breaking the belts attached to it, I removed it, and placed him upon his back upon a buffalo robe upon the floor, and setting down beside him I placed a ball of wick yarn in his axilla, and drew my boot and placed my heel upon it, and seizing his wrist with both my hands at the same time directing an assistant to seize me by the shoulders from behind, and another to seize him in the same way, and so on till I had the force of four men, when at a given signal we all made gradual but continued extension for about three minutes, when the bone slipped into its place with an audible snap, and immediately the patient was relieved, and the free motions of the arm were restored. The arm and hand were very much swollen, and he complained of great numbness of the entire limb, with the total loss of the power to contract his fingers, but this I attributed to the pressure of the pad in the axilla upon the vessels and nerves of the arm ; but after having applied such remedies as reduced the swelling, I discovered a dislocation of two of the bones of the carpus, but the inflammation and consequent adhesions were such as to forbid any attempt at reduction (if indeed that is possible under any circumstances,) and to destroy almost entirely the use of the hand. He has at this date very free use of the arm, but very little of the hand.

CASE V. I was called to see Thomas D., a miner, 30 years of age, about five feet ten inches high, and very large and muscular, at 8 o'clock, P. M., Sept. 21, 1852. On examination I found a dislocation of the femur directly backwards upon the dorsum ilii. He was suffering a good deal of pain, and upon comparing the two bones of the thigh, the dislocated one was from two to three inches shorter than the other, and immoveably fixed. The history he gave of his case was, that as he was coming out of the mines he stopped to talk to some one, and while doing so he leaned his back against one of the gangway timbers, and placed his left foot upon the bumper of an empty car that was standing on the track, and while in that position a loaded car came down the track and struck the car upon which he had his foot, and carried it forward with such force as to flex his leg suddenly upon his thigh, and thereby strike his knee, and his back being firmly fixed against the gangway timber offered so much resistance that the head of the femur was carried directly backward into the position in which I found it. I secured a staple into the surface upon one side of the house, and into the door casing upon the other, each being about three feet from the floor. I then placed a bed upon the floor and placed the patient upon the sound side upon it, and applied the compound pulleys in the usual way, and kept up extension and counter extension for nearly an hour with two men at the pulleys, when the patient positively refused to let me go on with my efforts, unless I put him under the influence of chloroform. But having very lately read of a number of cases terminating fatally from the use of chloroform in the hands of those who were in the habit of useing it constantly, I thought it best to have a consultation before proceeding further. I therefore gave the patient an anodyne, and directed him not to be moved, and adjourned the case until nine o'clock the next day.

At the time appointed, Dr. Carpenter met me in consultation, and I applied the pulley in the usual way, except that I applied the extending band to the ankle, instead of applying it immediately above the knee, as is usual, in consequence of the excessive developement of the muscles of the thigh, which made the link so tapering near the knee as to render it impossible to keep the extending band in position, while applying the necessary force. I now set the patient up in bed, with all the apparatus properly applied, and tied up his arm and made a very large orifice, and let the blood flow till he was on the point of fainting; when I immediately arrested the flow of blood, and commenced administering the ether (thinking that less dangerous than chloroform) and as soon he was completely under its influence, we placed him in position and commenced gradual extension, but before we had made it sufficiently long, the staples gave way and we were obliged to desist. We now laid the patient down and sent for a carpenter, and had the staples so secured as to prevent their giving way, at the same time raising the one attached to counter extending band to almost five and a half feet from the floor, so as to make it bear in such a way, as to assist in lifting the head of the bone from its malposition. We again placed him completely under the influence of the ether, and put four men to the pulleys, and two to the transverse band, when I took charge of the lifting band, and Dr. Carpenter the leg. After applying our force very gradually and keeping up extension for about five minutes, the head of the bone slipped into its place, with a very loud snap, and in letting him down the motions of the limb were restored, and it corresponded in every respect with its fellow. I placed him in bed and applied a bandage, so as to confine the injured limb to the sound one, and in a few minutes (the effects of the ether having passed away,) the patient expressed his gratitude for relief, in the warmest terms. I gave him a liniment to be applied to his hip, and directed him to be kept quiet in bed for a few days. At the end of ten days he was walking about with a stick entirely restored except a feeling of weakness in the vicinity of the joint.

Case 6.—I was called to a neighbouring beer house, in a great hurry, Nov. 23, 1852, to see Thomas C., a boatman 25 years of age, stout, thick set and very muscular, whom I found very much intoxicated, and two or three of his comrades holding him to restrain him from injuring himself; and others around him. He had a silk handkerchief bound tightly around his right arm, and he was covered with blood, as was the floor, ceiling, and sides of the room, and indeed everything in it. The history of his case was, that some fifteen or twenty minutes previous, in attempting to strike one of his companions, he drove his arm through a pane of glass in the window, dividing the muscles of the forearm down to the bone, about the junction of the lower with the middle third; at the same time dividing the radial artery. He drew his arm back and drove it down a second time upon the sharp pieces still hold in the sash, when a small pointed piece penetrated the forearm about one inch above the first wound, aad divided the radial artery a second time. The blood was still flowing from under the handkerchief, which had been bound around the arm by the bystanders. I immediately placed the thumb of an assistant upon the brachial artery, directing him to hold it firmly till I returned, when I came home and procured my horse shoe tourniquet, and applied it over the artery, and had him removed to his own house. By the time he was taken home and placed in bed, he was so overcome with the loss of blood and the effects of the liquors that he offered no resistance whatever, but was as passive as a child. I placed him upon his back in bed, near the edge, and drew his arm out over the side, and had it held firmly by an assistant. I now made an incision obliquely over the course of the artery, about one inch above the upper wound; but in consequence of the tissues being bathed in blood, I found it very difficult to apply the ligature, but I finally succeeded; but as the flow of blood still continued, I was obliged to apply a ligature to the lower end, which I did in the large wound, without much trouble, and then brought the wounds intact by several stitches of the interrupted suture and adhesive straps. I also applied a bandage pretty tightly, commencing at the ends of the fingers, and as I passed up with the bandage I applied a compress over the wounds. I ordered perfect quiet, and cold water dressings as soon as reaction came on.

November 26th, 9 o'clock, A. M.—Patient rested pretty well till near morning, when he became restless and uneasy. He is now very restless and feverish, with a good deal of pain in his head and arm, pulse 112, tongue white, dry, and very thirsty. To have calomel ʒss., to be divided into three equal parts, one to be taken every two hours, and to be followed by senna and salts, in divided doses till it operates freely. 10 o'clock, P. M. The medicine has operated very freely, less pain in the head and arm, less thirst, but very nervous and restless. To have one fourth of a grain of the sulph. of morphia every hour until he rests well, and to have cold lemonade for a drink.

November 27th, 9 o'clock, A. M.—Patient passed a very good night. Better in every respect. Continue treatment. My patient from this time on continued to improve, the ligatures coming away on the tenth day, and by the end of the third week he had entirely recovered. He now experiences no inconvenience in using his arm whatever.

Case 7.—I was called to see Mary S——, aged seven years, a child with light hair, blue eyes, a very fair skin, and altogether of a very delicate make. At the time I was called she was laboring under a very high fever, face flushed, with a purple spot on each cheek, rapid breathing, short dry cough, pulse 120, tongue covered with a white fur, and very thirsty, skin hot and dry, and severe pain in the left breast.

*Physical Symptoms.*—Complete hepatisation of the left lung, as shown by a dull sound over the entire lung, and the absence of the healthy respiratory murmur, and the existence of tubal respiration. Decubitus always upon the left side. I bled her freely till near syncope, and gave her an antimonial emetic, to be followed by calomel in five grain doses every four hours, with mucilaginous lemonade for drink, and flying sinapisms to the chest and back.

November 24th.—Patient somewhat relieved, pulse still 120, but less corded, cough still dry, pain less acute, but still severe, skin still dry and husky, and the tongue covered with a yellowish fur, particularly near the root, and the physical signs much the same as yesterday. Blood drawn yesterday cupped and buffy, clot very firm. Emetic operated very freely, and caused her to throw up large quantities of tough white mucus, but no blood. The calomel has operated very freely upon the bowels. Continued calomel and antimony in small doses every ten hours. Flying sinapisms to the chest and back, and cold lemonade with gum acacia for drink.

November 25.—Patient is still very much the same, but more feeble in consequence of the bowels being very loose. Discontinue calomel and antimony, and to have iodide of potassium, three grains every four hours, with tinct. opii and camph. in the syr. sarsaparilla compound. A liniment of equal parts of the ol. terebin., acid acetic, and ol. olivæ, to be rubbed to the chest, and to be covered with carded cotton constantly. Dov. pulv. with the hyd. c. cretâ at bed time. Continue cold mucilaginous drinks.

November 26th.—Patient considerably improved, but the mother complains that she cannot give the medicine, as she is very obstinate, and spits it out as fast as it is administered. The same kind of treatment was ordered to be continued, but very little could be done, as she would not take the medicines, and the parents would not force her, as they had made up their minds that she was going to die, although I had assured them if they could get her to take her medicines I thought she would recover, and thus things continued up to the 6th of January, 1853; some days taking one dose of her medicine, some two, and some none; some days being a little better, and on others a great deal worse. I had not seen her since the 21st of December, as I had discontinued my visits in consequence of their not compelling her to take the medicines. I now found her laboring under all the symptoms of abscess of the lung. Pulse 130, small and weak, cheeks flushed, skin bathed with perspiration, particularly when she sleeps, bowels very loose, tongue clean, shining, and very red, and on examining the chest I found the dulness much the same as at last report, but in the intercostal spaces between the sixth and seventh, and seventh and eighth ribs, immediately below the axilla, I found a very considerable bulging, and I thought I could detect fluctuation. I ordered a warm poultice to be kept constantly to the side, and a continuance of the iodide of potassium and syr. sarsaparilla, with the hydr. c. cretâ at bed time. To have a good diet of animal broths, with a little wine or porter three times a day.

January 10th.—The enlargement in the intercostal spaces becoming more prominent. Patient in much the same condition as at last report, except that the appetite has improved some, but her breath is very offensive, and a few evenings since, in an effort at vomiting, she threw up a small quantity of very offensive pus. I now prepared to operate upon the child, and draw off the matter with a trocar, but this the parents resisted, and would not hear of such a thing. Continue treatment as before.

January 14th.—Patient still much the same as at last report, except that she is becoming exhausted more rapidly. Friends still resist an operation. Continue treatment as before, except that she is to have the ol. jecor. aselli, in teaspoonful doses every four hours, in porter, and the hyd. c. cretâ every four or six hours, as may be necessary to restrain the bowels.

January 20th. The disease has progressed much more rapidly towards a fatal termination since last report. Her breath has become so very offensive, that the family can scarce remain in the room with the door closed. Parents have now consented to an operation, they being satisfied that she must die in a very short time without an operation, and that she can but die with it. Continue the treatment, with the addition of the elixir of vitriol in ten drop doses, three times daily.

January 21st. In the presence of and with the assistance of Dr. J. Carpenter, after drawing the skin directly upwards and making it tense, I made a slight incision immediately over the upper edge of the 8th rib, and then passed the trocar and canula into the chest, and drew off about ten ounces of extremely offensive pus of a greenish color. The air passed in and out of the canula as she inspired and expired. Blood flowed freely from the wound after removing the canula, but ceased upon closing it. After closing the wound with adhesive straps, we applied a compress over it, and bandaged the entire chest from below upwards. She bore the operation very well, and seemed much relieved after it. Continue treatment.

January 26th. Diarrhœa nearly ceased, coughs but very little, breath much less offensive, appetite becoming very good, and she has become very fond of the oil. From this time forward, my patient progressed favorably till March, when the abscess partially filled again; but an opening formed with one of the bronchial tubes, and after a fit of coughing, she was seized with vomiting, and threw up near half a pint of the same kind of greenish pus as before, with perfect relief to all of her symptoms. Some time in April, the same thing took place, but to a much less extent. I examined her *lung* in July, when I found healthy respiration in all parts of the lung except that part occupied by the abscess, which is dull and the side contracted. I have seen her frequently in the last few days, and she appears to be perfectly restored so far as her delicate constitution will permit.

